# Supplementary material for: EnzML: multi-label prediction of enzyme classes using InterPro signatures
Source: BMC Bioinformatics. 2012 Apr 25;13:61. doi: 10.1186/1471-2105-13-61 (PMC3483700; doi:10.1186/1471-2105-13-61)
Supplement: Addtional file 5 — The Java code to format the data files, evaluate and predict. The file enzml_java_code.tar.gz contains the Java code used to format database data to ARFF and XML formats, to execute cross and train-test (jackknife) evaluations and to record evaluation results to database. More information is included in the readme.txt file and the Javadoc files. The code can be used with a MySQL database. To use a different database software, other JDBC drivers might be required. [file 1471-2105-13-61-S5.gz › java_code/utils/doc/uk/ac/ed/inf/utils/PropertiesUtils.html]

PropertiesUtils


---


|  |  |  |
| --- | --- | --- |
| **Overview** \| **Package** \| **Class** \| **Use** \| **Tree** \| **Deprecated** \| **Index** \| **Help** | |  |
| **PREV CLASS**   **NEXT CLASS** | **FRAMES**    **NO FRAMES**     **All Classes** |  |
| SUMMARY: NESTED \| FIELD \| CONSTR \| METHOD | DETAIL: FIELD \| CONSTR \| METHOD |  |


---


## uk.ac.ed.inf.utils Class PropertiesUtils

```
java.lang.Object
  uk.ac.ed.inf.utils.PropertiesUtils
```

---

``` public class PropertiesUtils extends java.lang.Object ```

Utilities to manipulate Java properties and properties text files

**Version:**
:   4 Jan 2008

**Author:**
:   Luna De Ferrari luna.deferrari-at-ed.ac.uk

---

**Constructor Summary**

`PropertiesUtils()`


| **Method Summary** | |
| --- | --- |
| `static void` | `getJavaSystemProperties()`             Gets the Java system properties |
| `static java.util.Vector<java.lang.String>` | `getKeysStrings(java.util.Properties props)`             Get the properties keys as strings |
| `static java.util.Vector<java.lang.String>` | `getValuesStrings(java.util.Properties props)`             Get the properties values as strings |
| `static java.util.Properties` | `readLocalProperties(java.lang.String resourceName)`             Reads properties that inherit from three locations. |
| `static java.util.Properties` | `readProperties(java.lang.String propertiesFilePath)`             Reads properties Code adapted from Weka: http://www.cs.waikato.ac.nz/ml/weka/ Ian H. |

**Methods inherited from class java.lang.Object**

`equals, getClass, hashCode, notify, notifyAll, toString, wait, wait, wait`

**Constructor Detail**

### PropertiesUtils

```
public PropertiesUtils()
```


**Method Detail**

### getJavaSystemProperties

```
public static void getJavaSystemProperties()
```

:   Gets the Java system properties

---


### getKeysStrings

```
public static java.util.Vector<java.lang.String> getKeysStrings(java.util.Properties props)
```

:   Get the properties keys as strings

    :   **Parameters:**: `props` - a properties object **Returns:**: a vector with one string for each key in the properties

---


### getValuesStrings

```
public static java.util.Vector<java.lang.String> getValuesStrings(java.util.Properties props)
```

:   Get the properties values as strings

    :   **Parameters:**: `props` - a properties object **Returns:**: a vector with the properties values as strings

---


### readLocalProperties

```
public static java.util.Properties readLocalProperties(java.lang.String resourceName)
                                                throws java.lang.Exception
```

:   Reads properties that inherit from three locations. Code from Weka:
    http://www.cs.waikato.ac.nz/ml/weka/
    Ian H. Witten and Eibe Frank (2005)
    "Data Mining: Practical machine learning tools and techniques", 2nd
    Edition, Morgan Kaufmann, San Francisco, 2005.
    Reads properties that inherit from three locations. Properties are first
    defined in the system resource location (i.e. in the CLASSPATH). These
    default properties must exist. Properties defined in the users home
    directory (optional) override default settings. Properties defined in the
    current directory (optional) override all these settings.

    :   **Parameters:**: `resourceName` - the location of the resource that should be loaded. e.g.: "weka/core/Utils.props". (The use of hardcoded forward slashes here is OK - see jdk1.1/docs/guide/misc/resources.html) This routine will also look for the file (in this case) "Utils.props" in the users home directory and the current directory. **Returns:**: the Properties **Throws:**: `java.lang.Exception` - if no default properties are defined, or if an error occurs reading the properties files.

---


### readProperties

```
public static java.util.Properties readProperties(java.lang.String propertiesFilePath)
                                           throws java.lang.Exception
```

:   Reads properties Code adapted from Weka:
    http://www.cs.waikato.ac.nz/ml/weka/
    Ian H. Witten and Eibe Frank (2005)
    "Data Mining: Practical machine learning tools and techniques", 2nd
    Edition, Morgan Kaufmann, San Francisco, 2005.

    :   **Returns:**: the Properties **Throws:**: `java.lang.Exception` - if no default properties are defined, or if an error occurs reading the properties files.


---


|  |  |  |
| --- | --- | --- |
| **Overview** \| **Package** \| **Class** \| **Use** \| **Tree** \| **Deprecated** \| **Index** \| **Help** | |  |
| **PREV CLASS**   **NEXT CLASS** | **FRAMES**    **NO FRAMES**     **All Classes** |  |
| SUMMARY: NESTED \| FIELD \| CONSTR \| METHOD | DETAIL: FIELD \| CONSTR \| METHOD |  |


---
